# Timing of resting zone parathyroid hormone-related protein expression affects maintenance of the growth plate during secondary ossification: a computational study

**DOI:** 10.1007/s10237-024-01899-3

**Published:** 2024-11-16

**Authors:** Jorik Stoop, Yuka Yokoyama, Taiji Adachi

**Affiliations:** 1 Wallace H. Coulter Department of Biomedical Engineering, Georgia Institute of Technology and Emory University, Atlanta, GA 30332 USA; 2 Department of Biosystems Science, Institute for Life and Medical Sciences, Kyoto University, 53 Shogoin-Kawahara-Cho, Sakyo-Ku, Kyoto, 606-8507 Japan; 3 Department of Micro Engineering, Graduate School of Engineering, Kyoto University, 53 Shogoin-Kawahara-Cho, Sakyo-Ku, Kyoto, 606-8507 Japan; 4 Department of Mammalian Regulatory Network, Graduate School of Biostudies, Kyoto University, 53 Shogoin-Kawahara-Cho, Sakyo-Ku, Kyoto, 606-8507 Japan; 5 Department of Medicine and Medical Science, Graduate School of Medicine, Kyoto University, 53 Shogoin-Kawahara-Cho, Sakyo-Ku, Kyoto, 606-8507 Japan

**Keywords:** Secondary ossification, Growth plate, Bone development, Computational biomechanics, PTHrP

## Abstract

Secondary ossification and maintenance of the growth plate are crucial aspects of long bone formation. Parathyroid hormone-related protein (PTHrP) has been implicated as a key factor in maintaining the growth plate, and studies suggest that PTHrP expression in the resting zone is closely related with formation of the secondary ossification center (SOC). However, details of the relationship between resting zone PTHrP expression and preservation of the growth plate remain unclear. In this study, we aim to investigate the role of resting zone PTHrP expression on maintenance of the growth plate using a computational method. We extend an existing continuum-based particle model of tissue morphogenesis to include PTHrP and Indian hedgehog (Ihh) signaling, allowing the model to capture biochemical and mechanical regulation of individual cell activities. Our model indicates that the timing of resting zone PTHrP expression—specifically the rate of increase in production at the onset of SOC formation—is potentially a crucial mechanism for maintenance of the growth plate.

**Supplementary Information:**

The online version contains supplementary material available at 10.1007/s10237-024-01899-3.

## Introduction

Most bones form by the process of endochondral ossification, in which cartilage is gradually replaced by bone. Endochondral ossification involves the proliferation, differentiation, and hypertrophy of chondrocytes and is tightly regulated by various signaling pathways (Kronenberg 2003; Provot and Schipani 2005). During endochondral ossification of long bones, two ossification centers form and are essential for determining the overall shape and structure of the bone. First, in the center of the diaphysis, chondrocytes hypertrophy and initiate development of the primary ossification center (POC). The POC progresses longitudinally, with growth occurring at each end due to cell proliferation and hypertrophy in the growth plates. Later, the secondary ossification center (SOC) initiates in the epiphysis, separating the growth plate and articular cartilage.

While many major mechanisms of primary ossification have been elucidated, less is known about mechanisms regulating secondary ossification. One phenomenon without a clear mechanistic explanation is the maintenance of the growth plate between the primary and secondary ossification centers during development. Parathyroid hormone-related protein (PTHrP) is an important regulator that has been suggested as a key factor in growth plate maintenance (Hirai et al. 2011; Wysolmerski 2012). During endochondral ossification, PTHrP prevents chondrocyte differentiation and subsequent hypertrophy (Kronenberg 2003; Ohba 2020). Experimental studies have shown that PTHrP is expressed in the resting zone of the growth plate—the layer of resting chondrocytes above the proliferative zone—during secondary ossification, suggesting that negative regulation of PTHrP on chondrocyte hypertrophy may prevent destruction of the resting zone by delaying the approach of differentiating chondrocytes from the SOC above and POC below (Chen et al. 2007, 2008).

Additional findings have provided more insight into the function of resting zone chondrocytes during secondary ossification. Experiments using clonal genetic tracing in mice revealed that formation of the SOC causes formation of a stem cell niche in the growth plate where resting zone chondrocytes start to renew themselves (Chagin and Newton 2019; Newton et al. 2019). Another study indicated that these skeletal stem cells form in the resting zone from PTHrP-positive chondrocytes (Mizuhashi et al. 2018). Interestingly, results showed low numbers of PTHrP-positive chondrocytes in the resting zone before SOC initiation and increasing numbers of PTHrP-positive chondrocytes in the resting zone as the SOC expanded, suggesting a fundamental relationship between SOC formation and activation of PTHrP (Mizuhashi et al. 2018; Hallet et al. 2019). These findings indicate that the timing of PTHrP expression in the resting zone is related to development of the SOC, which may provide insight into the role of resting zone PTHrP expression in maintaining the growth plate.

To investigate the relationship between resting zone PTHrP expression and growth plate maintenance, we developed a computational model to simulate secondary ossification and predict the effect of changes in resting zone PTHrP expression. Other computational models of secondary ossification have implemented alternative assumptions to maintain the growth plate in their simulations. One model assumes that mechanical forces maintain the growth plate, with formation of the SOC protecting cartilage in the growth plate underneath from shear stresses and preventing ossification there (Sadeghian et al. 2021). Additionally, some models of secondary ossification have used reaction–diffusion equations to predict high PTHrP concentration in the lower epiphysis, leading to preservation of the chondrocytes in this area (Garzón-Alvarado et al. 2009, Peinado-Cortés et al. 2011). However, no previous model has considered the change in resting zone PTHrP expression shown to occur with SOC formation as a mechanism for maintaining the growth plate. In this study, we seek to better understand the relationship between resting zone PTHrP expression and maintenance of the growth plate during secondary ossification.

## Methods

### Continuum-based particle model

**Fig. 1:**
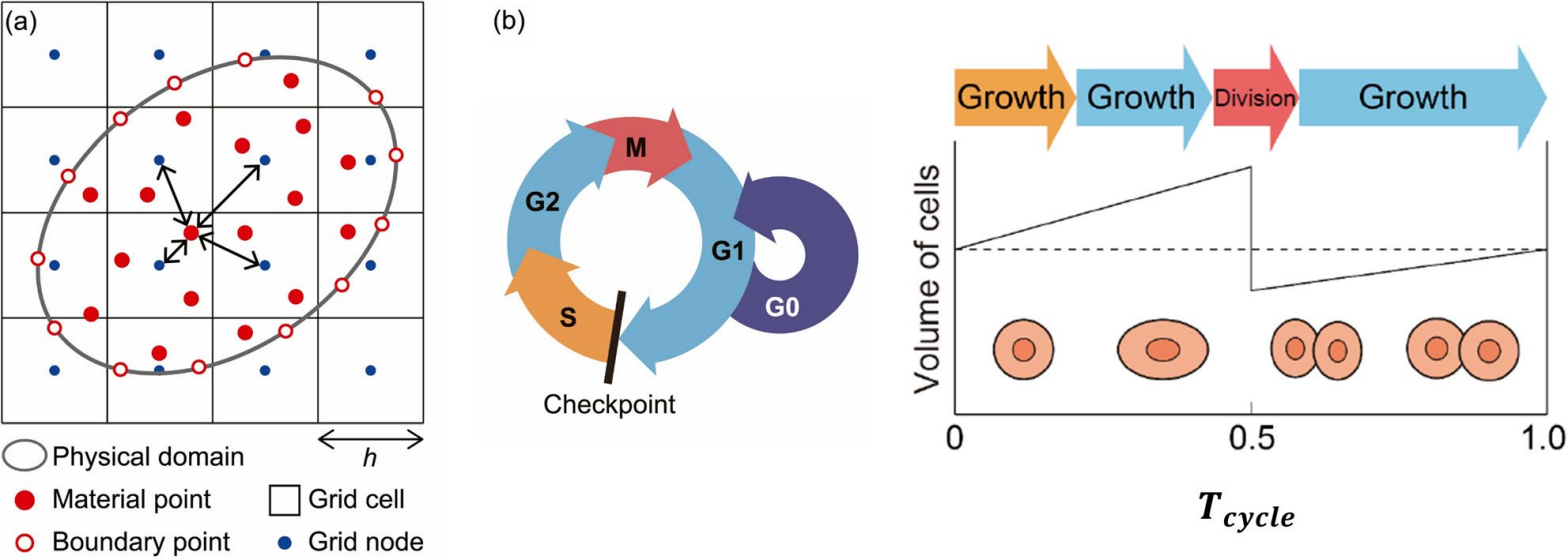
Concept of continuum-based particle method. **a** Representation of physical domain composed of material points and boundary points in MPM. Double sided arrows represent the interaction between a material point and the surrounding grid nodes. Parameter *h* determines grid spacing. **b** Cell cycle consisting of S (DNA replication), G2, M (mitosis), G1, and G0 (quiescence) phases. Growth occurs during S, G2, and G1 phases. Cell division occurs halfway through the cell cycle during M phase. Adapted from Yokoyama et al. (2023). Copyright https://s100.copyright.com/AppDispatchServlet?publisherName=ELS&contentID=S1751616123001819&orderBeanReset=true

Our continuum-based particle model (CbPM) is an extension of the model developed by Yokoyama et al. (2023) and is based on the material point method (Bardenhagen and Kober 2004). The material point method (MPM) is a hybrid method that combines advantages of both Lagrangian and Eulerian methods by using discrete Lagrangian material points to represent the physical domain and using Eulerian background grid nodes to solve the continuum mechanics-based equilibrium equations (Fig. 1a). Each material point possesses a position vector ${\varvec{x}}_{{\text{p}}}$, deformation gradient tensor ${\varvec{F}}_{{\text{p}}}$, and volume ${\varvec{V}}_{{\text{p}}}$. The discrete Eulerian grid nodes are used to solve for displacement $\varvec{u}\left( \varvec{x} \right)$ at any position ${\varvec{x}}$ by taking the displacement vectors of surrounding grid nodes ${\varvec{u}}_{\text{g}}$ and the interpolation function for those grid nodes $N_{{\text{g}}} \left( x \right)$:

1 $${\varvec{u}}\left( {\varvec{x}} \right) \approx \mathop \sum \limits_{\text{g}} {\varvec{u}}_{\text{g}} N_{\text{g}} \left( x \right)$$

A graphical representation of the interaction between a material point and its surrounding grid nodes is shown with double-sided black arrows in Fig. 1a. Quadratic extended B-splines are used as the interpolation function $N_{{\text{g}}} \left( x \right)$ for numerical stability (Yamaguchi et al. 2021), and the background grid spacing *h* is set to 50 μm (Yokoyama et al. 2023).

Here we consider a material point as a single cell and its surrounding matrix so that we can explicitly represent cell growth and division. To capture the tissue’s mechanical behavior, the bone tissue is assumed to obey the compressible neo-Hookean model (de Rooij and Kuhl 2018), and cell growth is modeled based on the theory of finite growth (Himpel et al. 2005; Takeda et al. 2020). For a given material point p, the deformation gradient ${\varvec{F}}_{{\text{p}}}$ is composed of both an elastic and growth component:

2 $${\varvec{F}}_{{\text{p}}} = {\varvec{F}}_{{\text{p}}}^{{\text{e}}} {\varvec{F}}_{{\text{p}}}^{{\text{g}}}$$

The constitutive equation for strain energy density $\psi$ based on the neo-Hookean assumption is expressed as:

3 $$\psi = J^{{\text{g}}} \left( {\frac{\lambda }{8}{\text{ln}}^{2} I_{3} + \frac{\upmu }{2}\left( {I_{1} - 3 - \ln I_{3} } \right)} \right)$$

where $J^{{\text{g}}}$ is Jacobian of ${\varvec{F}}_{{\text{p}}}^{{\text{g}}}$, $\lambda$ and $\upmu$ are Lamé parameters, and $I_{1}$ and $I_{3}$ are the first and third invariants of the elastic part of the right Cauchy–Green tensor, ${\varvec{C}}_{{\text{p}}}^{{\text{e}}} = {\varvec{F}}_{{\text{p}}}^{{{\text{eT}}}} {\varvec{F}}_{{\text{p}}}^{{\text{e}}}$. The Lamé parameters can be expressed using Young’s modulus *E* and Poisson’s ratio $\nu$, as $\lambda = \frac{E\nu }{{\left( {1 + \nu } \right)\left( {1 - 2\nu } \right)}}$ and $\upmu = \frac{E}{{2\left( {1 + \nu } \right)}}$ (Yokoyama et al. 2024). The deformation of the tissue is solved at discrete time increments, with a new position, deformation gradient, and volume calculated for each material point at increments of $\Delta t = 0.02$ days. Heterogenous cellular activities can be modeled by classifying material points as different cell types with distinct behaviors and mechanical properties. In this manner, individual cell activity is connected to the mechanical behavior of the whole tissue.

### Cell growth and proliferation

In this model, cell growth and proliferation are cell-type dependent. Proliferative chondrocytes in the growth plate are organized into columns, represented in our model by unidirectional growth, while prehypertrophic and hypertrophic cells undergo isotropic growth. Both unidirectional and isotropic cell growth are implemented for each material point by defining the growth part of the deformation gradient ${\varvec{F}}_{{\text{p}}}^{{\text{g}}}$ as a function of growth stretch $\theta$, as described in Yokoyama et. al (2024). Isotropic growth is expressed as:

4 $${\varvec{F}}_{{\text{p}}}^{{\text{g}}} = \theta {\varvec{F}}_{{{\text{p}}0}}^{{\text{g}}}$$

where ${\varvec{F}}_{{{\text{p}}0}}^{{\text{g}}}$ is the growth part of the deformation gradient before isotropic growth begins. Unidirectional growth is expressed as:

5 $${\varvec{F}}_{{\text{p}}}^{{\text{g}}} = \left( {{\varvec{I}} + \left( {\theta - 1} \right){\varvec{n}}_{{\text{s}}} { } \otimes {\varvec{n}}_{{\text{s}}} } \right){\varvec{F}}_{{{\text{p}}0}}^{{\text{g}}}$$

where the direction of cell growth ${\varvec{n}}_{{\text{s}}}$ is the same as the direction of cell division, ${\varvec{F}}_{{{\text{p}}0}}^{{\text{g}}}$ is the growth part of the deformation gradient before unidirectional growth begins, and ${\varvec{I}}$ is the second-order identity tensor.

To model cell proliferation, the state of each cell is classified into S (DNA replication), G2, M (mitosis), G1, and G0 (quiescence) phases (Fig. 1b). Only proliferative chondrocytes or prehypertrophic chondrocytes that enter S phase prior to differentiation undergo mitosis. In M phase, mitosis is modeled as a division of the material point into two new material points halfway through the cell cycle at time $t = 0.5\times T_{{{\text{cycle}}}}$ where $t = 0$ is the onset of S phase and $T_{{{\text{cycle}}}}$ is the time duration of S, G2, M, and G1 phases (Fig. 1b). The two new material points formed from cell division are created along the direction of cell division ${\varvec{n}}_{{\text{s}}}$, with one new material point appearing in the +${\varvec{n}}_{{\text{s}}}$ direction and the other in the $- {\varvec{n}}_{{\text{s}}}$ direction. The direction of division ${\varvec{n}}_{{\text{s}}}$ is determined by the gradient of Indian Hedgehog (Ihh) concentration, based on similar methodology used in previous computational studies (Vaca-González et al. 2018). The volume of each new material point is assumed to be half of the original point’s volume. After a cell cycle is completed, the cell goes into the quiescent G0 phase to wait for the onset of the next cell cycle. The length of the G0 phase is assumed to follow an exponential distribution with the rate parameter $R_{{{\text{prolif}}}}$:

6 $$T_{{{\text{G}}0}} = - \log \left( {1.0 - r} \right)/R_{{{\text{prolif}}}}$$

where $r$ is a randomly generated number between 0 and 1 (Yokoyama et al. 2023).

### Biochemical signaling

The biochemical factors Ihh and PTHrP regulate cell proliferation and differentiation in our model. Unlike a previous CbPM study of bone development which only includes one general differentiation factor (Yokoyama et al. 2024), this study explicitly models both Ihh and PTHrP signaling. During endochondral ossification, Ihh is produced by prehypertrophic and early hypertrophic cells (Kronenberg 2003; Provot and Schipani 2005). Therefore, we assume that Ihh production $P_{{{\text{Ihh}}}}$ $\left( {{\text{pM}}/{\upmu \text{m}}^{3} {\text{s}}} \right)$ increases to a maximum value $P_{{{\text{Ihh}},{\text{ max}}}}$ as prehypertrophic cells mature, and then subsequently decreases to zero halfway through hypertrophy:

7 $$\frac{{dP_{{{\text{Ihh}}}} }}{dt} = \left\{ {\begin{array}{*{20}l} {P_{{{\text{Ihh}},{\text{max}}}} /T_{{{\text{prehyp}}}} } \hfill & {\left( { t_{{\text{H}}} \le T_{{{\text{prehyp}}}} } \right)} \hfill \\ { - P_{{{\text{Ihh}},{\text{max}}}} /\frac{{T_{\text{hyp}} }}{2}} \hfill & {\left( {T_{{{\text{prehyp}}}} < t_{{\text{H}}} \le \frac{{T_{{\text{hyp}}} }}{2} + T_{{{\text{prehyp}}}} } \right)} \hfill \\ 0 \hfill & {\left( {else} \right)} \hfill \\ \end{array} } \right.$$

where $t_{{\text{H}}}$ is the time duration that a particular cell has been in the prehypertrophic or hypertrophic state, $T_{{{\text{prehyp}}}}$ is the duration that a cell remains in the prehypertrophic state before becoming hypertrophic, and $T_{{\text{hyp}}}$ is the duration that a cell remains in the hypertrophic state before undergoing apoptosis or turning into calcified matrix.

Ihh and PTHrP act as a negative feedback loop. Ihh signals to perichondrial cells and chondrocytes in the periarticular region to stimulate PTHrP expression, while PTHrP in turn prevents columnar proliferating chondrocytes from differentiating into Ihh-producing prehypertrophic cells (Provot and Schipani 2005; Wysolmerski 2012). The mechanism by which Ihh signals to the periarticular region to stimulate PTHrP production is not fully understood but may be explained by intermediate signaling through TGF-beta and direct signaling (Alvarez et al. 2002; Hilton et al. 2007; Wysolmerski 2012). It has been suggested that Ihh affects PTHrP on a concentration dependent manner (Ohba 2020), therefore PTHrP production $P_{{{\text{PTHrP}}}}$ $\left( {{\text{pM}}/{\upmu}\text{m}^{3} {\text{s}}} \right)$ by periarticular chondrocytes in the model is expressed as:

8 $$P_{{{\text{PTHrP}}}} = P_{{{\text{PTHrP}},{\text{max}}}} \frac{{Ihh_{{{\text{total}}}} }}{{Ihh_{{{\text{max}}}} }}$$

where $P_{{{\text{PTHrP}},{\text{max}}}}$ is the maximum possible PTHrP production, $Ihh_{{{\text{total}}}}$ is the total amount $\left( {{\text{pM}}} \right)$ of Ihh in the tissue at the current moment in the simulation, and $Ihh_{{{\text{max}}}}$ is a constant used to normalize the value. $Ihh_{{{\text{max}}}}$ is calculated by taking the maximum total amount of Ihh in a sample simulation of endochondral ossification.

Here we assume that resting zone PTHrP production coincides with development of the SOC. At the time of the SOC onset, a region of quiescent cells above the proliferative zone of the growth plate and below the SOC is defined as the resting zone. In resting zone chondrocytes, PTHrP production increases over time as the SOC matures:

9 $$ P_{{{\text{PTHrP}}}} = \left\{ {\begin{array}{*{20}l} {P_{{{\text{PTHrP}},{\text{max}}}} \,\, \frac{{Ihh_{{{\text{total}}}} }}{{Ihh_{{{\text{max}}}} }} \,\, \frac{{t_{{{\text{SOC}}}} }}{{T_{{{\text{mat}}}} }} } \hfill & {(t_{{{\text{SOC}}}} < T_{{{\text{mat}}}} )} \hfill \\ {P_{{{\text{PTHrP}},{\text{max}}}} \,\, \frac{{Ihh_{{{\text{total}}}} }}{{Ihh_{{{\text{max}}}} }}} \hfill & {\left( {t_{{{\text{SOC}}}} \ge T_{{{\text{mat}}}} } \right)} \hfill \\ \end{array} } \right. $$

where the variable $t_{{{\text{SOC}}}}$ is the time duration from the onset of SOC formation and $T_{{{\text{mat}}}}$ is a scalar value defining the time at which the SOC has matured enough for resting zone cells to produce normal levels of PTHrP. This reflects the increase in resting zone PTHrP production observed during SOC formation in experimental results (Mizuhashi et al. 2018). In the model, the rate of increase of PTHrP production is determined by the $T_{{{\text{mat}}}}$ parameter. Resting zone PTHrP expression reaches the same expression levels as in the periarticular region once $t_{{{\text{SOC}}}} \ge T_{{{\text{mat}}}}$.

The diffusion of Ihh and PTHrP are described by:

10 $$\frac{{\partial C_{{{\text{Ihh}}}} }}{\partial t} = D\nabla^{2} C_{{{\text{Ihh}}}} + P_{{{\text{Ihh}}}} - kC_{{{\text{Ihh}}}}$$

11 $$\frac{{\partial C_{{{\text{PTHrP}}}} }}{\partial t} = D\nabla^{2} C_{{{\text{PTHrP}}}} + P_{{{\text{PTHrP}}}} - kC_{{{\text{PTHrP}}}}$$

where $C_{{{\text{Ihh}}}}$ and $C_{{{\text{PTHrP}}}}$ are the concentrations $\left( {{\text{pM}}/{\upmu \text{m}}^{3} } \right)$ of Ihh and PTHrP respectively, $D$ is the diffusion coefficient, $P_{{{\text{Ihh}}}}$ and $P_{{{\text{PTHrP}}}}$ are the production of each biological factor, and $k$ is the degradation rate. In previous studies that simulated reaction and diffusion of PTHrP and Ihh during bone development, the diffusion coefficients had values between $1.1 \times 10^{ - 19}$ and $1.1 \times 10^{ - 9} {\text{m}}^{2} /{\text{s}}$ (Garzón-Alvarado et al. 2009; Peinado Cortés et al. 2011). Here we assume that both Ihh and PTHrP have the same diffusion coefficient $D = 1.0 \times 10^{ - 12} {\text{ m}}^{2} /{\text{s}}$, which is within the range used in previous studies, and the same degradation rate $k$, which is arbitrarily set to $k = 0.005 /s$. The assumption that both chemicals have the same diffusion coefficient and degradation rate was made due to the proteins’ similar size of 19 kDa for the N-terminal peptide of Ihh (Ohba 2020) and 18 kDa for PTHrP (Soki et al. 2012). The concentrations of Ihh and PTHrP are both set to 0 sufficiently far from the tissue. As described in Yokoyama et al. (2024), symmetrical boundary conditions are applied to the planes *x* = 0, *y* = 0, and *z* = 0 for diffusion analysis.

### Cell differentiation

By considering discrete material points as individual cells, the CbPM connects heterogenous cell activities to overall tissue shape and mechanics. In this model, each material point is defined as either quiescent, proliferating, prehypertrophic, hypertrophic, apoptotic, matrix, perichondrium, or bone collar. PTHrP producing cells are defined using separate cell types to allow for spatially specific PTHrP expression. PTHrP-producing cells in the resting zone are otherwise identical to quiescent cells, while PTHrP-producing periarticular cells are otherwise identical to perichondrial cells.

Differentiation is regulated by Ihh and PTHrP thresholds (Table 1). Threshold Ihh levels determine differentiation from quiescent to proliferative cells. The concentration of Ihh for a quiescent material point must surpass the threshold concentration $C_{{{\text{th Ihh}},{\text{prolif}}}}$ to become proliferative. The transition from the proliferative to prehypertrophic cell type is regulated by both Ihh and PTHrP. A given material point must be above the Ihh concentration threshold $C_{{{\text{th Ihh}},{\text{prehyp}}}}$ and below the PTHrP concentration threshold $C_{{{\text{th PTHrP}},{\text{prehyp}}}}$ to differentiate. After differentiation, a prehypertrophic cell will change cell type to hypertrophic after a fixed time $T_{{{\text{prehyp}}}}$. During bone morphogenesis, primary spongiosa forms as mature hypertrophic chondrocytes undergo apoptosis and the surrounding matrix becomes calcified by osteoblasts. Our model captures this process by having hypertrophic cells either undergo apoptosis or turn into calcified matrix with equal probability (50% chance apoptosis and 50% chance matrix) after time $T_{{{\text{prehyp}}}} + T_{{\text{hyp}}}$. To reflect the change in material properties as chondrocytes are replaced by calcified matrix, the Young’s modulus *E* and Poisson’s ratio $\nu$ of the material points gradually change during time duration $T_{\text{apop}}$ for apoptosis and $T_{\text{calcif}}$ for calcification, as described by Yokoyama et al. (2024). The model does not take bone remodeling into account, so the apoptotic and matrix cell types remain unchanged for the duration of the simulation. Lastly, Ihh regulates the differentiation of the perichondrium into bone collar (Kronenberg 2003), so material points with perichondrial cell type change into bone collar if their Ihh concentration surpasses the threshold concentration $C_{{{\text{th Ihh}},{\text{BC}}}}$.

**Table 1:** Thresholds regulating cell differentiation

Cell type transition	Threshold for differentiation
Resting chondrocytes → proliferative	$C_{{{\text{Ihh}}}} > C_{{{\text{th Ihh}},{\text{prolif}}}}$
Proliferative → prehypertrophic	$C_{{{\text{Ihh}}}} > C_{{{\text{th Ihh}},{\text{prehyp}}}} \, \& \& \, C_{{{\text{PTHrP}}}} < C_{{{\text{th PTHrP}},{\text{prehyp}}}}$
Prehypertrophic → hypertrophic	$t_{{\text{H}}} > T_{{{\text{prehyp}}}}$
Hypertrophic → primary spongiosa	$t_{{\text{H}}} > T_{{{\text{prehyp}}}} + T_{{\text{hyp}}}$
Perichondrial → bone collar	$C_{{{\text{Ihh}}}} > C_{{{\text{th Ihh}},{\text{ BC}}}}$

The Ihh threshold concentration for proliferative differentiation, $C_{{{\text{th Ihh}},{\text{prolif}}}} = 1.0 {\text{ pM}}/{\upmu \text{m}}^{3}$, and the Ihh threshold for prehypertrophic differentiation, $C_{{{\text{th Ihh}},{\text{prehyp}}}} = 30.0 {\text{ pM}}/{\upmu \text{m}}^{3}$, were determined through one-dimensional simulation of the growth plate described in Yokoyama et al. (2024) to produce proliferative and hypertrophic zones of reasonable size, with a combined length of $200 - 400 \, {\upmu \text{m}}$ (Reno et al. 2006). The PTHrP threshold concentration for inhibiting prehypertrophic differentiation, $C_{{{\text{th PTHrP}},{\text{prehyp}}}} = 0.5 {\text{ pM}}/{\upmu \text{m}}^{3}$, was determined through simulations of primary ossification that reproduced a reasonably sized zone of undifferentiated subarticular cartilage of $\sim 30 \, {\upmu \text{m}}$. The Ihh threshold concentration for bone collar formation, $C_{{{\text{th Ihh}},{\text{BC}}}} = 10.0 {\text{ pM}}/{\upmu \text{m}}^{3}$, was determined by Yokoyama et al. (2024) such that calcified bone collar is observed near the hypertrophic zone. A list of model parameters is provided in Table 2.

**Table 2:** Model parameters

Symbol	Description	Value	Reference
*Biochemical signaling*			
$D$	Diffusion coefficient of PTHrP and Ihh	1.0 ${\upmu}\text{m}^{2} /s$	Garzón-Alvarado et al. (2009); Peinado-Cortés et al. (2011)
$k$	Degradation rate constant of PTHrP and Ihh	0.005 $/s$	Yokoyama et al. (2024)
$P_{{{\text{PTHrP,max}}}}$	Maximum value of PTHrP production	1.0 ${\text{pM}} / {\upmu}\text{m}^{3} s$	arbitrary
$P_{{{\text{Ihh,max}}}}$	Maximum value of Ihh production	1.0 ${\text{pM}} / {\upmu}\text{m}^{3} s$	arbitrary
$Ihh_{{{\text{max}}}}$	Constant for normalizing PTHrP production	1.5 $\times 10^{6} {\text{pM}}$	Section 2.3
*Cell differentiation and proliferation*			
$C_{{{\text{th Ihh}},{\text{prolif}}}}$	Threshold Ihh concentration for proliferative differentiation	1.0 ${\text{pM}}/ {\upmu}\text{m}^{3}$	Section 2.4
$C_{{{\text{th Ihh}},{\text{prehyp}}}}$	Threshold Ihh concentration for prehypertrophic differentiation	30.0 ${\text{pM}}/ {\upmu}\text{m}^{3}$	Section 2.4
$C_{{{\text{th PTHrP}},{\text{prehyp}}}}$	Threshold PTHrP concentration for prehypertrophic differentiation	0.5 ${\text{pM}}/ {\upmu}\text{m}^{3}$	Section 2.4
$C_{{{\text{th Ihh}},{\text{BC}}}}$	Threshold Ihh concentration for bone collar formation	10.0 ${\text{pM}}/ {\upmu}\text{m}^{3}$	Section 2.4
$T_{\text{cycle}}$	Time duration of the cell cycle	1.0 day	Hayflick et al. (1961)
$T_{\text{prehyp}}$	Time duration of prehypertrophy	0.5 day	Yokoyama et al. (2024)
$T_{\text{hyp}}$	Time duration of hypertrophy	1.0 day	Farnum et al. (2008)
$T_{\text{apop}}$	Time duration of apoptosis	1.0 day	Yokoyama et al. (2024)
$T_{\text{calcif}}$	Time duration of calcification	1.0 day	Yokoyama et al. (2024)
$T_{\text{mat}}$	Time duration of SOC maturation	0.5–16 days	
$R_{\text{prolif}}$	Rate parameter of proliferation	5.0 /day	Yokoyama et al. (2024)
*Cell mechanical properties*			
$E_{\text{cell}}$	Young’s modulus of chondrocytes	1.0 kPa	Luo et al. (2016)
$E_{\text{calcif}}$	Young’s modulus of bone matrix	$1.0 \times 10^{3}$ kPa	Yokoyama et al. (2024)
$E_{\text{BC}}$	Young’s modulus of bone collar	$1.0 \times 10^{2}$ kPa	arbitrary
$v_{\text{cell}}$	Poisson’s ratio of chondrocytes	0.4	Trickey et al. (2006)
$v_{\text{calcif}}$	Poisson’s ratio of bone matrix	0.3	Pidaparti and Vogt (2002)
$v_{\text{BC}}$	Poisson’s ratio of bone collar	0.3	Pidaparti and Vogt (2002)

In this study, we incorporate a novel feature into the CbPM by simulating secondary ossification. While cell differentiation in the POC and SOC are regulated by the same biochemical signals, initiation of the primary and secondary ossification centers follows separate assumptions. For initiation of the POC, a layer of cells spanning the center of the diaphysis are set to the prehypertrophic cell type. For initiation of the SOC, a cluster of cells in the epiphysis are set to the prehypertrophic cell type later in development ($t_{{{\text{SOC}}}} = 0)$.

### Initial conditions

**Fig. 2:**
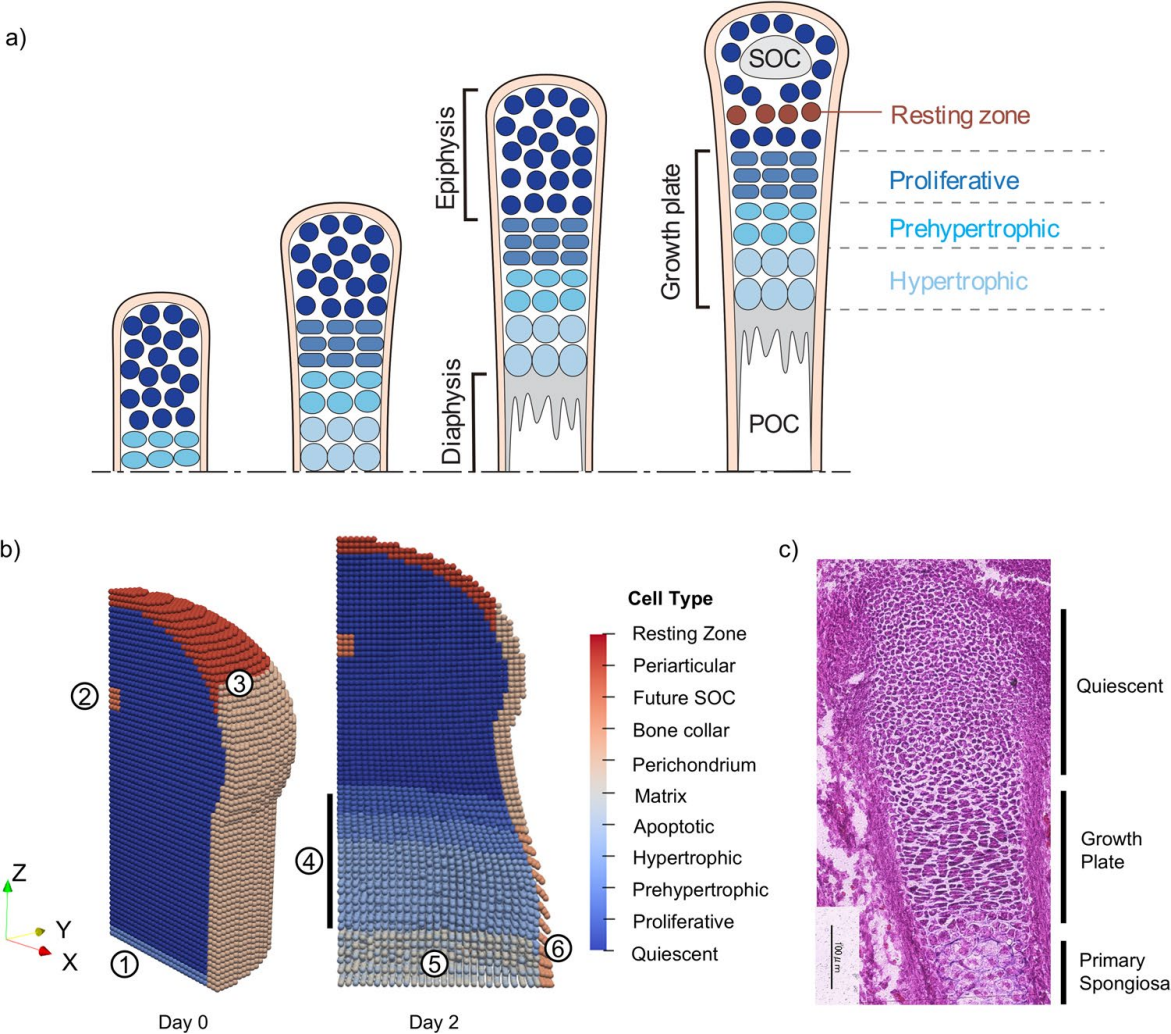
Progression of endochondral ossification in CbPM and mouse metatarsal. **a** Illustration of a growing long bone from the onset of primary ossification. As the growth plate develops, it separates the epiphysis and diaphysis. Later in development the SOC forms in the epiphysis and the resting zone forms above the proliferative cells in the growth plate. **b** Progression of primary ossification in the CbPM: (1) Cells in the diaphysis are initially defined as prehypertrophic and initiate primary ossification. (2) A group of cells in the center of the epiphysis are designated to later become prehypertrophic and initiate secondary ossification. (3) Cells at the edge of the bone are designated as perichondrium or PTHrP producing periarticular cells. (4) Growth plate develops into proliferative, prehypertrophic, and hypertrophic zones. (5) Primary spongiosa forms consisting of matrix and apoptotic cells. (6) Bone collar forms near the hypertrophic zone. **c** Metatarsal bone of E16 (embryonic day 16) mouse with labels showing the location of quiescent cells, growth plate, and primary spongiosa. Hematoxylin and Eosin staining was conducted as described by Yokoyama et al. (2024)

The distal portion of a mouse metatarsal bone was modeled due to its simple and axisymmetric shape. The 3D geometry for the initial bone shape was created by combining a cylinder with radius 300 μm and height 300 μm and a dome of height 340 μm with an *x*- and *y*- radius of 330 μm and *z*-radius of 250 μm. The bottom of the center axis of the cylinder was considered the origin (*x* = 0,* y* = 0, and* z* = 0), with the *z*-axis set as the longitudinal axis of the cylinder and the *x*- and *y*-axes set perpendicular to the *z*-axis. One quarter of the distal metatarsal bone, or one-eighth of the whole tissue, was modeled assuming mirror symmetry on the *x* = 0,* y* = 0, and* z* = 0 planes. The resulting 3D bone capsule was composed of 42,861 regularly distributed material points with a spacing of 10 μm and 5,491 boundary points (Fig. 2b). The initial volume of each material point was set to $V_{{\text{p}}} = 10^{3} \, {\upmu \text{m}}^{3}$. Slip boundary conditions were applied for the* x* = 0,* y* = 0, and* z* = 0 planes.

## Results

### Resting zone PTHrP production is essential for growth plate preservation in secondary ossification

Using the constructed model of cell growth, proliferation, and differentiation, simulations of endochondral ossification were conducted in 3D. On Day 0, primary ossification is initiated and cell types including the perichondrium, PTHrP producing cells in the periarticular region, and cells designated as future SOC are defined (Fig. 2b). As the simulation progresses, prehypertrophic cells become hypertrophic and Ihh production leads to differentiation of nearby quiescent cells into proliferative cells. Cell hypertrophy and proliferation drive growth and cells differentiate into regions of proliferative, prehypertrophic, and hypertrophic cells characteristic of the growth plate (Fig. 2a, b). A histological image of an E16 (embryonic day 16) mouse metatarsal bone is included to highlight how growth plate formation in the simulation closely aligns with experimental observation (Fig. 2c). In the E16 metatarsal bone, the epiphysis is comprised of quiescent chondrocytes, a growth plate is identifiable with layers of proliferative, prehypertrophic, and larger hypertrophic cells, and the primary spongiosa is shown below the growth plate (Fig. 2c). These features qualitatively agree with the cell types and their locations in the model on Day 2 (Fig. 2b). Furthermore, in histological images of an E14 mouse metatarsal from Yokoyama et al. (2024), the bone tissue is composed entirely of quiescent chondrocytes and perichondrium, similar to the initial condition of the model at Day 0 (Fig. 2b).

**Fig. 3:**
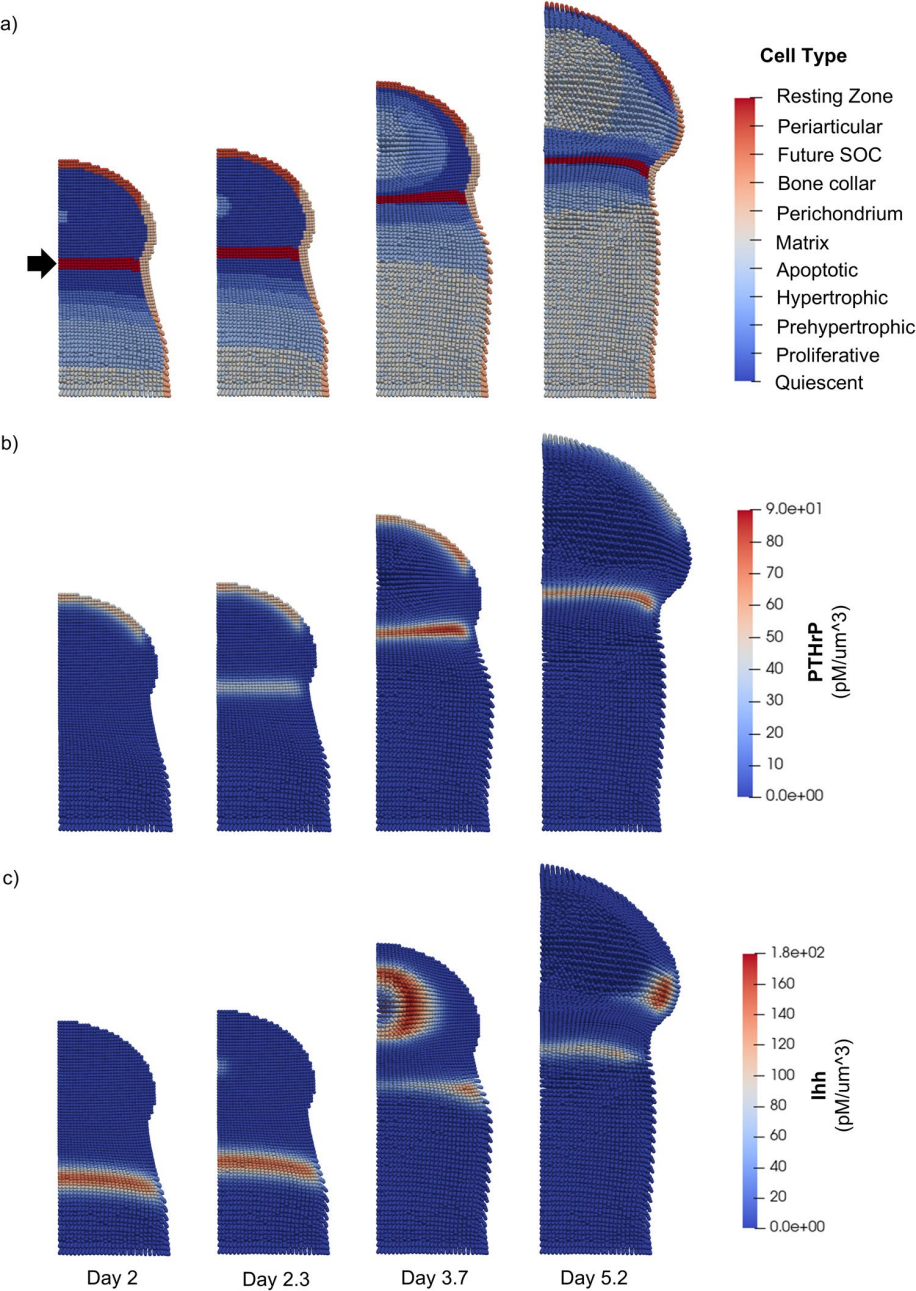
Simulation of secondary ossification with resting zone PTHrP expression. 2D cross-sectional views of the bone capsule show tissue structure and signaling from Day 2 to Day 5.2. **a** Cell types. The solid arrow indicates the resting zone which forms above the growth plate at the onset of SOC formation. **b** PTHrP concentration values. PTHrP expression occurs in periarticular cells and in the resting zone. **c** Ihh concentration values. Ihh expression occurs in the prehypertrophic and hypertrophic zones of the growth plate and SOC. The total amount of Ihh modulates expression of PTHrP

To show that resting zone PTHrP expression can maintain the growth plate during development, endochondral ossification was simulated starting from the onset of primary ossification through secondary ossification (Fig. 3a–c, Supplementary Video 1). In the model, Ihh produced by prehypertrophic and hypertrophic cells (Fig. 3c) causes nearby quiescent chondrocytes to become proliferative, allowing the growth plate to advance and increase the length of the bone. As the growth plate reaches the epiphysis, secondary ossification is initiated at Day 2 $(t_{{{\text{SOC}}}} = 0)$ and a region of quiescent cells below the SOC is defined as the resting zone (Fig. 3a). PTHrP production in the resting zone increases over time as the SOC expands (Fig. 3b). As secondary ossification progresses, the SOC initially expands radially. Later, the SOC expands in a hemispherical direction as differentiation of the lower edge is inhibited by PTHrP produced in the resting zone (Fig. S1). Importantly, the growth plate is maintained throughout the simulation. Resting zone PTHrP production in the model keeps the locations where the PTHrP threshold value is reached at a sufficient distance from the resting zone to prevent encroachment of hypertrophic cells from the SOC above and the POC below.

The change in shape of the SOC from spherical to hemispherical (Fig. 3a, Supplementary Video 2), agrees with previous experimental studies of epiphysial ossification (Álvarez et al. 2005; Kwong et al. 2014). In the study by Álvarez et al. (2005) using rat tibia, epiphyseal polarity developed between postnatal day 11 and 14. Ossification continued unchanged at the proximal side facing the articular surface, but was attenuated at the distal side, causing the SOC shape to change from spherical to hemispherical (Álvarez et al. 2005). The 3-day period of SOC shape change observed in the rat tibia is similar to the time scale of SOC shape change in the model, which occurs between Days 2.3 and 5.2 (Fig. 3a). Additionally, SOCs in human humerus bones have demonstrated a transition in shape from oval to hemispheric (Kwong et al. 2014), demonstrating that this structural change is common across different types of bones. The simulation demonstrates qualitative agreement with the structure and time scale of development of the hemispherical SOC shape in vivo.

**Fig. 4:**
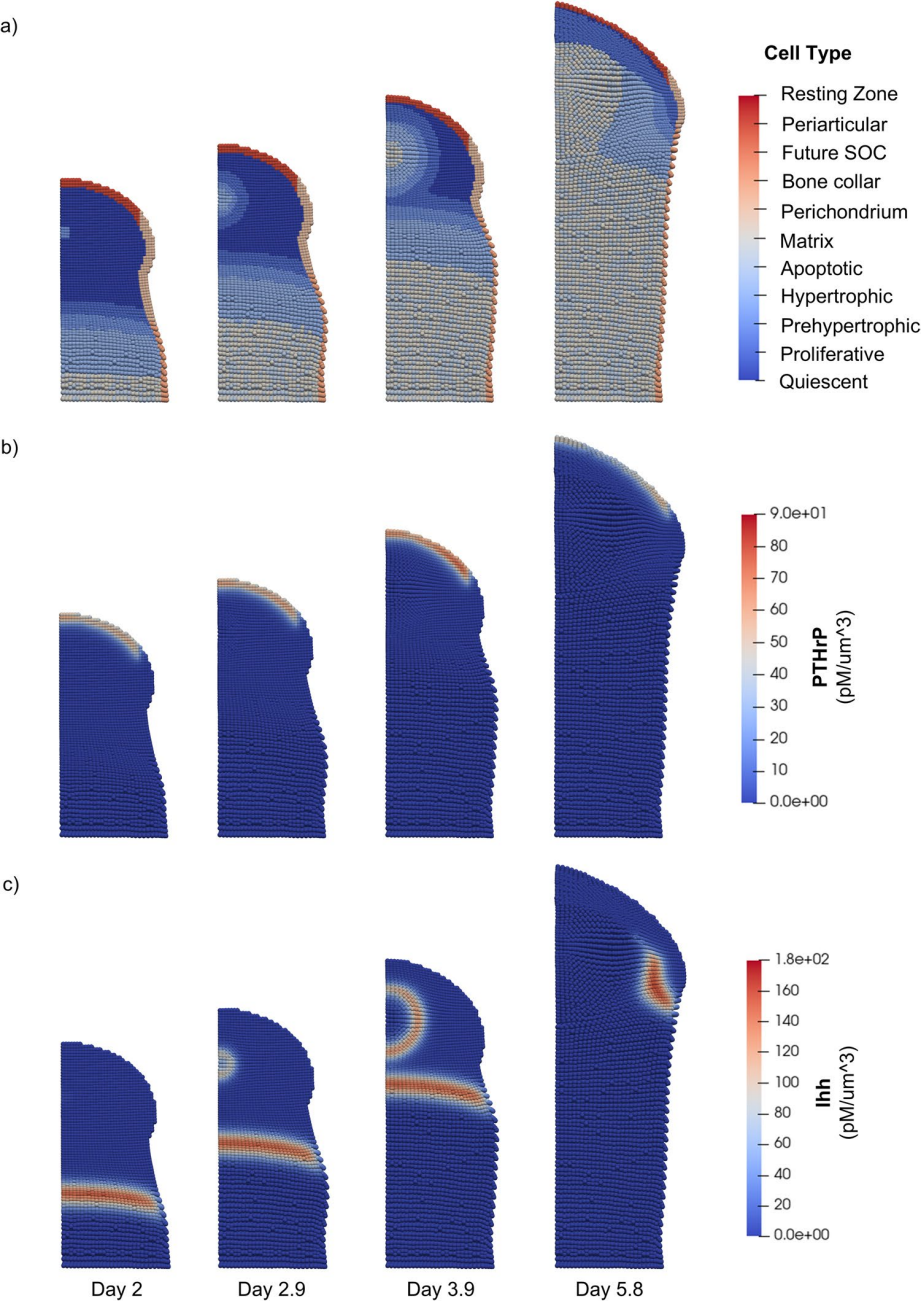
Simulation of secondary ossification with no resting zone PTHrP expression. 2D cross-sectional views of the bone capsule show tissue structure and signaling from Day 2 to Day 5.8. **a** Cell types. There is no resting zone defined at the onset of secondary ossification. The zones of calcified bone from both the SOC and POC consisting of matrix and apoptotic cells are fused at Day 5.8 **b** PTHrP concentration values. PTHrP expression occurs in the periarticular perichondrium. **c** Ihh concentration values. Ihh expression occurs in prehypertrophic and hypertrophic zones. The total amount of Ihh modulates expression of PTHrP

Furthermore, when secondary ossification was simulated without any PTHrP production in the resting zone, early fusion of the growth plate was observed (Fig. 4a, Supplementary Video 3). In this simulation, as the growth plate reaches the epiphysis and secondary ossification is initiated, no PTHrP-producing resting zone region is defined (Fig. 4a). The absence of PTHrP expression in the resting zone (Fig. 4b) allows the prehypertrophic fronts of both the primary and secondary centers to advance unopposed, resulting in collision of the SOC and growth plate and eventual fusion of the two ossification centers. Ihh production is also affected by the fusion of the growth plate. In the case without growth plate fusion, Ihh production initially occurs in the growth plate (Day 2 in Fig. 3c), then increases in the epiphysis as prehypertrophic and hypertrophic cells form (Day 3.7 in Fig. 3c), and later decreases as hypertrophic cells in the epiphysis become ossified (Day 5.2 in Fig. 3c). In the case with growth plate fusion, Ihh production also initially occurs in the growth plate, increases in the epiphysis after onset of the SOC, and later decreases as hypertrophic cells in the epiphysis are ossified (Fig. 4c). However, as the SOC and POC collide, the distinct prehypertrophic and hypertrophic zones of the growth plate are lost, eliminating Ihh production in that area (Day 5.8 in Fig. 4c), whereas Ihh production in the growth plate is retained in the simulation with resting zone PTHrP production (Day 5.2 in Fig. 3c). Taken together, the results of these two cases indicate that resting zone PTHrP production is important for maintaining the growth plate during simulations of secondary ossification.

### Rate of increase in resting zone PTHrP expression after onset of SOC determines whether growth plate is maintained

**Fig. 5:**
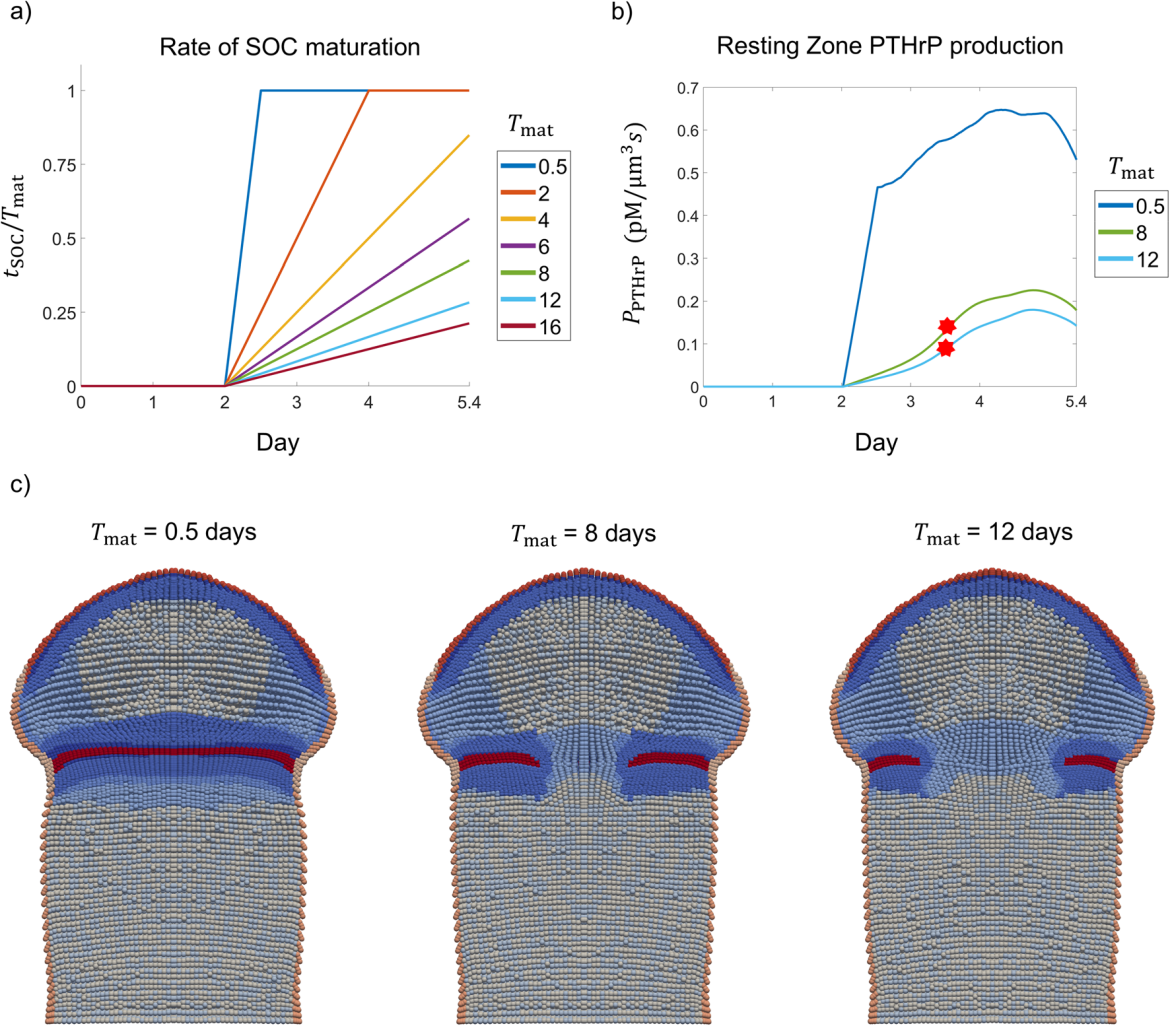
Effect of resting zone PTHrP production rate on growth plate preservation. **a** Parameter $T_{{{\text{mat}}}}$ modulates the rate of SOC maturation. The graph displays the slope of SOC maturation over time for various values of $T_{{{\text{mat}}}}$, with shorter maturation times having steeper slopes. **b** Resting zone PTHrP production calculated from three simulations with varying $T_{{{\text{mat}}}}$. Stars indicate the time point of growth plate fusion. **c** Mirrored 2D cross-sections of the bone capsule show tissue structure at Day 5.4 in simulations with $T_{{{\text{mat}}}} = 0.5, 8, 12 {\text{ days}}$. Penetration of the resting zone (red) is observed for simulations with longer maturation times ($T_{{{\text{mat}}}} \ge 8 {\text{ days}}$) and slower PTHrP production rates

Next, we conducted simulations with varied rates of PTHrP production to investigate the relationship between the timing of resting zone PTHrP expression and growth plate preservation (Fig. 5). For these simulations, resting zone PTHrP expression was initiated at the onset of secondary ossification and the rate of increase of PTHrP production was varied by modulating $T_{{{\text{mat}}}}$, which defines the time duration until the SOC is considered mature. Once the SOC is defined as mature, PTHrP production by resting zone chondrocytes is equivalent to that of periarticular chondrocytes. In the model, the fraction $t_{{{\text{SOC}}}} /T_{{{\text{mat}}}}$ controls the rate of resting zone PTHrP production (Eq. 9, Fig. 5a). The actual values of resting zone PTHrP production in the simulation increase with rates similar to the slope of $t_{{{\text{SOC}}}} /T_{{{\text{mat}}}}$, and fluctuate later in the simulation (Fig. 5b). The fluctuation in level of PTHrP production can be explained by changes in the total amount of Ihh, which increases and then decreases along with the number of prehypertrophic cells as the SOC expands (Fig. S2).

Simulations were conducted with $T_{{{\text{mat}}}}$ values ranging from 0.5 to 16 days. Each simulation was run for 5.4 days with resting zone PTHrP production beginning when secondary ossification is initiated at Day 2. In simulations with faster SOC maturation, the growth plate was maintained throughout the simulation. Specifically, when $T_{{{\text{mat}}}} \le 6 {\text{ days}}$ and the slope of $t_{{{\text{SOC}}}} /T_{{{\text{mat}}}}$ was greater than or equal to 0.168 per day, resting zone PTHrP production rates were sufficient to prevent growth plate fusion. In simulations with longer SOC maturation times ($T_{{{\text{mat}}}} \ge 8 {\text{ days}}$), resting zone PTHrP production increased slowly (Fig. 5b) and penetration of proliferative and hypertrophic chondrocytes into the resting zone was observed (Fig. 5c, Supplementary Video 4). These results suggest that there is a critical value of $T_{{{\text{mat}}}}$ over which PTHrP production is too slow and PTHrP does not diffuse far enough to prevent the SOC and POC from invading the resting zone. Therefore, our model predicts that a sufficiently quick increase of resting zone PTHrP expression after the onset of secondary ossification is necessary to prevent differentiation in the resting zone and maintain the growth plate.

## Discussion

We have developed a model of bone morphogenesis using a continuum-based particle method. By modeling cell growth, proliferation, and differentiation in a cell-type dependent manner, we account for spatially heterogenous cell activity and connect this to the behavior of the entire tissue. The addition of Ihh and PTHrP signaling to the CbPM framework makes it possible to study the effects of both biochemical signaling and mechanical influences on tissue structure. Our model demonstrates a potential mechanism for growth plate maintenance where an inhibitory PTHrP signal produced in the resting zone prevents prehypertrophic differentiation and preserves the growth plate. Our model predicts that resting zone PTHrP production must increase quickly at the onset of secondary ossification to prevent fusion of the primary and secondary ossification centers.

Previous experimental studies have shown that Ihh/PTHrP signaling is essential for maintaining the growth plate throughout development. Both postnatal ablation of Ihh and postnatal knockout of the PTH/PTHrP receptor (PPR) resulted in premature growth plate fusion (Maeda et al. 2007; Kimura et al. 2008; Hirai et al. 2011). In the study by Hirai et al. (2011), deletion of the PPR was performed in mouse tibia at postnatal day 3. Premature growth plate fusion occurred between 3 and 7 days after PPR knockout, while in the control mice with active PPRs the growth plates were maintained between the SOC and POC (Hirai et al. 2011). In our simulation with no resting zone PTHrP production, the lack of PTHrP in the resting zone is similar to the chondrocyte-specific PPR knockout because both result in a loss of PTHrP signaling in the growth plate. The simulation results qualitatively agree with the experimental observations since the simulation without resting zone PTHrP expression reproduces premature growth plate fusion and the control simulation with resting zone PTHrP expression maintains the growth plate.

Additional studies have described the formation of a stem cell niche of PTHrP-positive chondrocytes in the resting zone closely related to formation of the SOC (Mizuhashi et al. 2018; Newton et al. 2019), providing a possible mechanism for previous suggestions that PTHrP expression in resting zone chondrocytes plays a role in maintaining the growth plate. We found that PTHrP production by resting zone chondrocytes starting at SOC onset results in preservation of the growth plate only when PTHrP production increases at a sufficient rate. In this model, resting zone PTHrP expression prevents nearby chondrocytes from becoming hypertrophic where PTHrP concentrations are above the threshold for differentiation. Thus, the POC and SOC cannot penetrate the resting zone and the growth plate is maintained. However, when resting zone PTHrP production increases too slowly, PTHrP is not able to diffuse far enough to prevent hypertrophic differentiation before the proliferative fronts of the POC and SOC breach the resting zone. Therefore, the production and diffusion rates are important parameters for growth plate maintenance in our model, suggesting that the rate of resting zone PTHrP production must be sufficiently fast to preserve the growth plate.

Our model demonstrated that the rate of resting zone PTHrP production is an important parameter for maintaining the growth plate with our current implementation of Ihh and PTHrP signaling. Therefore, we identify a possible mechanism for growth plate preservation. However, further study with alternative implementations of Ihh and PTHrP signaling, as well as validation of parameter values against in vivo experimental results remains necessary to predict the key factors needed for maintaining the growth plate. Experiments characterizing the diffusion rates and expression levels of Ihh and PTHrP in the growth plate during secondary ossification would be especially useful. Additionally, to better understand the relationship between SOC formation and activation of resting zone PTHrP expression, future studies of biochemical or mechanical factors linking the two may be interesting. One idea is that as the SOC expands, diffusion of Sonic hedgehog—another member of the hedgehog protein family—from the SOC and diffusion of Ihh from prehypertrophic cells in the growth plate cause the resting zone to have especially high hedgehog signaling (Newton et al. 2019). Ihh signaling is known to increase PTHrP production, so this may contribute to the specific location of PTHrP expression in the resting zone.

Additionally, it has been shown that the SOC protects chondrocytes in the growth plate from mechanical stress (Xie et al. 2020), which was used to explain growth plate preservation in a previous computational model of endochondral ossification (Sadeghian et al. 2021). In two studies that account for external forces, model results indicated that low octahedral shear stresses contribute to preventing hypertrophy and maintaining cartilage in the growth plate (Peinado Cortes et al. 2011; Sadeghian et al. 2021). Although our model does not account for external loading, the simulation results qualitatively agree with these other models by reproducing growth plate preservation during secondary ossification. The transition in SOC shape to a hemispherical form in our model is also in agreement with the work by Peinado Cortes et al. (2011), where hypertrophy in the epiphysis changed orientation from radial to hemispheric. However, these studies do not account for changes in resting zone PTHrP expression during secondary ossification. By extending our model to include external forces, we could explore the effects of mechanical stresses or mechanosensing on resting zone PTHrP expression and help determine the degree to which mechanical and biological factors each contribute to growth plate preservation.

In summary, we developed a computational framework to study secondary ossification and investigated how the timing of resting zone PTHrP production affects growth plate maintenance. Our CbPM incorporates heterogenous cell activity and combines biochemical and mechanical regulation to predict changes in overall tissue behavior, making it a powerful tool for modeling complex tissue morphogenesis. Using this method, we simulated development of the POC and SOC and predicted that a sufficient rate of resting zone PTHrP production is potentially a crucial mechanism for maintaining the growth plate.

## Supplementary Information

Below is the link to the electronic supplementary material.

Supplementary file1 (PDF 332 KB)

Supplementary file2 (MP4 26711 KB)

Supplementary file3 (MP4 5142 KB)

Supplementary file4 (MP4 25959 KB)

Supplementary file5 (MP4 9707 KB)

## Data Availability

All data needed to evaluate the conclusions in the paper are present in the paper. Additional data related to this paper may be requested from the authors. Source codes related to this paper may be requested from the authors.

## Code availability

Source codes related to this paper may be requested from the authors.
