# Supplementary material for: Timing of resting zone parathyroid hormone-related protein expression affects maintenance of the growth plate during secondary ossification: a computational study
Source: Biomech Model Mechanobiol. 2024 Nov 16;24(1):125–37. doi: 10.1007/s10237-024-01899-3 (PMC11846766; doi:10.1007/s10237-024-01899-3)
Supplement: Supplementary file 1 — Supplementary file1 (PDF 332 KB) [file 10237_2024_1899_MOESM1_ESM.pdf]

**Supplementary material**

**Title:** Timing of resting zone parathyroid hormone-related protein expression affects maintenance of the growth plate during secondary ossification: a computational study.

**Journal:** Biomechanics and Modeling in Mechanobiology

**Authors:** Jorik Stoop[a], Yuka Yokoyama[b,c], Taiji Adachi[b,c,d,e]

**Affiliations:**

[a] Wallace H. Coulter Department of Biomedical Engineering, Georgia Institute of Technology and Emory University, Atlanta, GA 30332, USA.

[b] Department of Biosystems Science, Institute for Life and Medical Sciences, Kyoto University
53 Shogoin-Kawahara-cho, Sakyo-ku, Kyoto, 606-8507, Japan.

[c] Department of Micro Engineering, Graduate School of Engineering, Kyoto University
53 Shogoin-Kawahara-cho, Sakyo-ku, Kyoto, 606-8507, Japan.

[d] Department of Mammalian Regulatory Network, Graduate School of Biostudies, Kyoto University
53 Shogoin-Kawahara-cho, Sakyo-ku, Kyoto, 606-8507, Japan.

[e] Department of Medicine and Medical Science, Graduate School of Medicine, Kyoto University
53 Shogoin-Kawahara-cho, Sakyo-ku, Kyoto, 606-8507, Japan.

**Corresponding author:**
Taiji Adachi, Ph.D., Professor
Email address: adachi@infront.kyoto-u.ac.jp

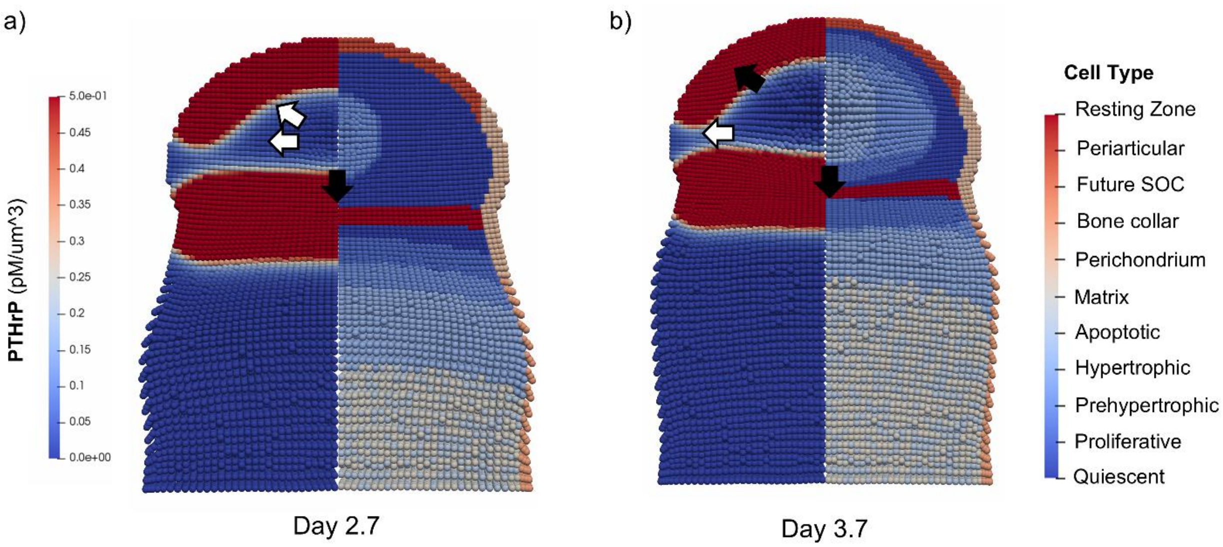


**Fig. S1** Inhibition of SOC at the distal edge of the epiphysis by resting zone PTHrP. 2D cross-sectional views of the bone capsule show PTHrP concentration values and cell types. Split views of PTHrP concentration and cell type are displayed at **(a)** Day 2.7, during early secondary ossification and **(b)** Day 3.7, later in SOC formation. The color bar for PTHrP concentration is set to a maximum of 0.5 $\mathrm{pM/\mu m^3}$ so that all material points above the PTHrP threshold for prehypertrophic differentiation ($C_{\mathrm{th\ PTHrP,prehyp}} = 0.5\ \mathrm{pM/\mu m^3}$) are colored red. White arrows indicate where the prehypertrophic zone of the SOC has room to expand, while black arrows indicate where the prehypertrophic zone of the SOC is impeded by PTHrP signaling.

As shown in Fig. S1, the proliferative material points at the bottom edge of the SOC are prevented from differentiating due to PTHrP concentration values over the threshold $C_{\mathrm{th\ PTHrP,prehyp}} = 0.5\ \mathrm{pM/\mu m^3}$. Early in secondary ossification, the proliferative fronts facing the sides of the epiphysis have room to expand due to adject material points having PTHrP concentration values below the threshold $C_{\mathrm{th\ PTHrP,prehyp}}$. This allows for expansion of the SOC in those directions while expansion towards the distal edge is attenuated, ultimately resulting in a hemispheric shape.

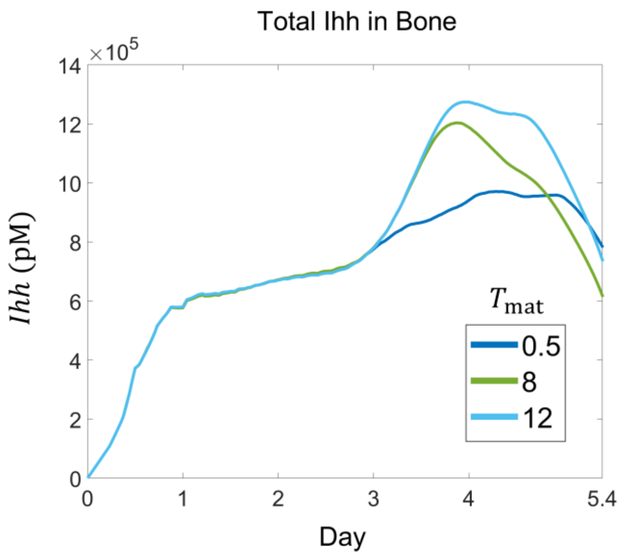


**Fig. S2** Total amount of Ihh for three simulations with varied resting zone PTHrP production rates. Total Ihh in the bone was calculated by summing the concentration of Ihh at each material point

The total amount of Ihh in the bone experiences a sharp increase around Day 3 (Fig. S2), approximately one day after secondary ossification is initiated at Day 2. This is likely due to the increase in prehypertophic and hypertrophic cells in the SOC that begin to produce Ihh. Similarly, an increase in resting zone PTHrP production is observed with a slight delay occurring between Day 3 and 4 (Fig. 5b), supporting the idea that changes in PTHrP production are driven by Ihh fluctuation in the simulation. Additionally, Ihh amounts peak before Day 4 for simulations with $T_{\mathrm{mat}} = 8, 12$ and decrease in Ihh in all three simulations after Day 4. This trend is also mirrored by the decrease in resting zone PTHrP production which occurs around Day 5 (Fig. 5b), further indicating that fluctuations in PTHrP production can be explained by changes in total Ihh.
